# Supplementary material for: MetaRibo-Seq measures translation in microbiomes
Source: Nat Commun. 2020 Jun 29;11:3268. doi: 10.1038/s41467-020-17081-z (PMC7324362; doi:10.1038/s41467-020-17081-z)
Supplement: Supplementary file 10 — Supplementary Data 7 [file 41467_2020_17081_MOESM10_ESM.zip › File2/Confidence_High_Taxonomy/299187_out.krona.html]

Javascript must be enabled to view this page.

members
magnitude
magnitudeUnassigned
count
unassigned
taxon
rank

299187\_out

3

2
superkingdom
3

3
phylum
976

class
3
200643

3
order
171549

815
family
3

3
genus
816

2292944

SRS893341\_contig\_number\_61
1
species

821

SRS056273\_contig\_number\_contig-100\_0.0
1
species

species
1
818

SRS075078\_contig\_number\_contig-100\_0.0
